# Modern Medical Journalism

**Published:** 1893-12

**Authors:** J. Greig Smith

**Affiliations:** Professor of Surgery in University College, Bristol, and Surgeon to the Bristol Royal Infirmary


THE BRISTOL

### Medico-Chirurgical Journal.

DECEMBER, 1893.

### MODERN MEDICAL JOURNALISM.

The Presidential Address, delivered on October 11th, 1893, at the opening of the 20th Session of the Bristol Medico-Chirurgical Society.

BY

J. Greig Smith, M.A, M.B., C.M. Aberd., F.R.S.E.,

*Professor of Surgery in University College, Bristol, and Surgeon to the Bristol Royal Infirmary.*

The opening sentences of a presidential address are, according to general and praiseworthy custom, devoted to expressions of thankful appreciation of the honour conferred and of gentle depreciation of the worthiness of the speaker. I desire to adhere to this good rule. I shall not dwell upon my unworthiness: it is not easy to be sincere about one's own failings. But the expression of pride in the honour may well be, and herewith is, fully outspoken. I am conscious of, and proud of, the dignity; and I trust that I shall not forget the responsibility as well. For in our Society, as elsewhere in our profession, ease does not come with dignity and honour, but rather increase of labour. I willingly and gladly accept the one with the other.

When a man so young as myself is elected to the most honourable office in your gift, it means either that some work has to be done of which you think he is capable, or that some reward is to be made which you think he has deserved; or, possibly, both causes exist. I take it that both causes do exist. The reward is for my editorship of our *Journal;* the work to be done relates to our Library. The one is, in a sense, the completion and fruition of the other; and, as I was present at the birth of the *Journal*, and had the sole responsibility of its early upbringing, perhaps it is fitting that I should continue to help forward the vigorous efforts of its adolescence. I do more than care for the success of the Library; my heart and soul are in it. I am, indeed, committed to it; for when the *Journal* began to earn money, and there was a likelihood of our generous committee distributing it too liberally to charities, I pleaded for the hoarding of it—that we might buy books and found a Library. These small hoardings accumulated, and they made possible, almost practicable, the existence of a Medical Library in our midst. The Medical School, the natural centre of our profession in this as in every other city which possesses a school, provides the admirable home for our Library where we are now met, and will at the same time make substantial additions in funds and in books. The valuable and extensive Infirmary Library will be placed on our shelves; the Hospital will lend its books; and it is an open secret that we may look forward to a valuable gift from a neighbouring institution. Associations, societies, and private individuals have generously added to our stores, and, we earnestly hope, will continue to do so. With some 10,000 volumes either on the shelves or very soon to be there, and some 90 current periodicals on the tables, we may fairly claim to have made a good start towards having a Medical Library worthy of our centre and of ourselves. But the work is little more than started. We have secured the grand fact of having all the smaller collections gathered into one large collection; we have provided for every medical man and for every medical student the freest possible access to our literature. We trust that this hall will be in the future the centre of our intellectual life in the West; and we hope that this life will derive a new strength and a higher value from nourishment on the pabulum collected within its walls.

And now I am going to talk to you for a little while on a subject which is germane to the motives which I believe have induced you to place me here; namely, "Modern Medical Journalism." Further reasons for my selection of subject are: that I have had something to do with medical journalism; that I have done with it; and that I have certain views about it which I should like you to hear and, if possible, to share.

Modern Journalism has certain characteristics which are peculiar to our age and time. This is the age of the reporter, not of the scholar. The material of literature is news, not thought; gossip, not work. The chatter of the newsmonger pushes aside the matured thought of the man of letters. We are deluged with a shallow flood of literature, and we are so occupied in paddling about in its smooth and tepid pools that we scarcely care to try our strength in the rougher and colder waters of deep thinking.

This is also the age of advertisement. The desire of the writer is rather to be widely read than to write well. The unavowed aim of the writer is the avowed aim of the journal—a wide circulation. And the purpose of this wideness of circulation is, not that knowledge may be diffused, but that advertisements may be caught—and money. Unknown men write to advertise themselves; known men are induced to write to advertise the journal. The chief end of journalism is to catch advertisements; the trail of the advertiser is over all.

Now, the population of these islands being, on good authority, mostly fools, the editor who hopes to secure the largest number of readers—the widest circulation—must cater accordingly. And so the successful editor does. The writing is not up to the highest intelligence, but down to the meanest capacity. The levelling is down—down to the dead level of the School Board. Free education, wide circulation, and cheapness—cheapness in every sense—go well together. They are the Fates—I had almost said the Furies—of Modern Journalism.

And now the advertiser is turning on us. He is already the mainstay of the press; naturally he seeks to become the arbiter of letters. It is openly stated that he now dictates to the editor what authors shall write for his paper. If a story by a certain popular author is not in the magazine, so increasing its circulation, the advertiser will withdraw his advertisement. It is whispered even that the review of a book or a report on manufactures may vary directly as the square of the inches occupied in advertising them. This is a sad fact for literature; but it is a fact, and we must face it. I confess that I should like to fight it.

Now, is there anything of this sort in Modern Medical Journalism? I should like to put to the modern medical editor one test question, and let his answer decide. I should ask: "Which would you consider the better copy for your journal—the clever gossip of a keen reporter hanging around the death-bed of an emperor, or the most scientific paper ever written on cancer of the larynx?" I know what his answer would be, and so do you.

There are sundry difficulties which meet the best-intentioned editor which he cannot remove, and which he must accept. One of these, I grieve to say, relates to the deficient literary education of the average medical man. For some mysterious reason the art and science of logic is not expected to be known by medical men. It is not known, and their writings show it. The arts of rhetoric and composition also are ignored; even the humdrum rules of grammar are in abeyance. How much of the editor's function is the humble one of correcting a school exercise in grammar and composition the editor only knows. It is certainly much more than it ought to be, and much more than it need be, if those who guard the portals of medical education were to give some heed to the outcry of those who practise their profession and teach it.

This, then, to begin with. We must accept the fact that, with a few, a very few, and sometimes brilliant exceptions, the literature of medicine at the present day is, as pure literature, either indifferent or positively bad. Scarcely a single issue of a journal is without a paper from which a man educated in ordinary literary methods could not extract abundant examples of errors in logic, in rhetoric, in composition, and even in humble grammar.

Another fact which the editor must accept is the widespread ignorance of the history of medicine—recent and remote—which his contributors display. Such ignorance, betraying itself on every hand, lends colour to the views of certain cynics who hold that it is only by courtesy that our profession is called "learned"; that we are educated by rote, and not by thought; that the evolution of ideas has no interest for us; and that our classics being dead, may as well be buried. There is ignorance not only of classics, but even of class-books. It would truly seem as if knowledge in our profession began to decay after five years, and was buried and forgotten after ten. However this may be, it is certain that too many writers show a culpable ignorance of, or neglect of, the works of others in the fields they seek to cultivate.

From this condemnation I desire to exclude the literature of our American brethren. The country which has given to us the *Index Medicus* and the *Index Catalogue* of its splendid library is not likely to fail in knowledge of the history of medicine. A knowledge of the work of others that is nearly always honest and thorough is as common in the best American medical literature as it is rare in our English journals. The man who writes on his own experience and knowledge writes on a poor and narrow and shifty foundation. In science he is to be found only in medicine; and in scientific medicine he bears the prefix —in all its significations—British.

It used to be true that the weight of the printed word was greater than of the spoken. We have changed all that. News is news none the less that it is either folly or lies: perhaps it circulates all the better. In past times also the printing of a book cost much money. Necessity, then, if nothing else, made a man think much before printing his thoughts. The value that a man put on the products of his brains was testified to by the finish of the garb in which he clothed them. It would be easy to put before you a score of books printed from a half to a whole century ago, and say of each of them that, in respect of printing, paper, binding, and more especially of illustrations, we never see their like now. The artistic finish then sought after is now replaced by the blurred mimicries of the "process block." And if we do borrow, as we frequently do, from the illustrations made by our predecessors, we treat them as time does their tombstones, by defacing their surfaces and roughening their lines; but not as time does, by making them venerable. What would a Cooper, or a Bell, or a Miller, or a Liston, or a Fergusson, be not justified in saying of the successive generations of book-makers who have borrowed their engravings and reproduced and defaced them?

And all that can be said in favour of our modern methods of illustration is that they are cheap. Certainly they are cheap.

And our journals! Look at those piles on our tables! Of the thousands and thousands of papers in them how many are worth preserving? how many are worth reading? how many are worth only cursing? The dew of knowledge! Medical knowledge is not a dew, but a deluge. It drowns rather than nourishes.

The man who earnestly and honestly seeks to keep abreast of real knowledge has to educate himself in a course of training in values whereby he finds out and knows the fool, and the drudge, and the bore, and the advertiser, and learns to avoid them. When he has done this, he finds that his labours are not so hard after all. It is, then, a mere turning over of thousands of leaves and a close reading of tens of pages. But it takes a long time, and greatly tries one's patience, to learn whom to avoid in medical literature.

And here our editors might help us. They might select for us. They might silence the chatterer and make the worker talk. You can scarcely put too cruel a gag on the garrulous. They are doubly mischievous, for not only do they waste the reading energies of their brethren, but they oust the work of men worth reading. It may be that the man who bustles about in the van of progress, seeming to do much but really doing nothing, like the Handy Andy of the circus, pleases the audience; and that may be why the editor supports him. But let us be careful lest the genuine actor in his dignity and pride, not caring to play to the gallery, and not asked to play to the stalls, wait outside altogether, or even retire to his tent and sulk.

The best men and the best workers are silent and retiring; the editor must seek them out, and drag them out, and make them speak. This is no easy matter, as I can from experience testify. It is very hard to get our best men to write. One of my failures I specially deplore, for the man whom I tried to persuade is now, to the great loss of the West of England, no more with us. I refer to the late William Liddon, of Taunton. His last promise to write for our *Journal* was given me between three and five one beautiful morning when we were driving over the Quantocks. I scarcely knew which most to admire: his vivid appreciation and description of a lovely sunrise then going on, or his exquisitely clear, almost photographic, language in describing our case and others in his experience that bore on it. He promised to write for our *Journal*: he might have done it; he cannot now. It is a loss to our art that the work of such men as Liddon lives only in the memories of friends and patients. It would be a gain if our editors were to insist on its being kept alive.

An equally great blessing would be to find some editors who, in respect of certain well-recognised classes of papers, would insist on their entombment.

There is the paper of the pushing clinician. We are warned of its appearance by a leaderette, which informs us that "at 1.30 on the afternoon of Tuesday, at the South London Hospital, Mr. Hepaticus Brown removed both supra-renals for Addison's disease. We are promised full particulars for a future number." The anxiously-awaited particulars appear a month later as a paper "On Removal of the Supra-renals for Addison's Disease: a successful case, with a complete list of all other operations." Hereafter Mr. Hepaticus Brown poses as the leading authority on diseases of the supra-renals. This path to high repute is much affected: it is easy and broad, if it is not very straight.

Then there is the pushing scientist. We, his teachers and examiners, probably knew him as a gentle, plodding young man with a great capacity for note-taking. Now, with the help of a scholarship, asked for and granted, he has started hotly in pursuit of something called Research (spelt with a capital letter). He tells us *everything* he sees in the new path, and he sees much that is wonderful. If such men could be made to hold their tongues until they had spent some years as hewers of wood and drawers of water in the workshops of our masters in Research, the world might be richer; it certainly would be none the poorer.

Then, once more, there is the pushing practitioner. His pride is puffed up and his reputation is increased by a systematic publication of all his wonderful cases. As we know, wonderful cases are found in greatest numbers by the youngest men in the smallest practices. Our pushing practitioner has not long left the schools; and he believes that science and art have stood still there since he left them. An editor who would give to such men the answer that the old lady gave to her pushing grandson when he sought to explain to her the philosophy of sucking eggs would be a gain to medical journalism.

And so on. It is weary work classifying the bores and the fools; and it would be wearier work crushing them. We are a polite people, we practitioners of medicine. Soothing the bodily pangs of suffering humanity seems to beget in us a sentimental horror of hurting the mental feelings of our worrying brethren. But let us not overdo it. Too much salaaming may make the spine too flexible; and we want backbone. To stand up squarely and strongly against all these abuses; to fight them and to crush them—who shall do it? It must be done if we seek to rise to higher levels. How are we to do it?

Here are our motives: to push aside the chatterer; to make the worker speak; to bring measures to the front, not men; to eschew all appearance of artificial advertisement, either for measures or for men; to let both measures and men rest on real merit; to speak the truth, and nothing but the truth, of men, of books, of papers, and of work; and throughout to have our purveyor of intellectual food like the high-class purveyor of material food, not only stand at the door and personally see that nothing inferior or unwholesome is distributed from his establishment, but also actively go out and seek for the best procurable pabulum for his customers. Such a man will not only stimulate the producer to surpass himself in invention; he will also crush out the incompetent and the careless. He is greatly wanted in our literary world.

May we hope for a higher medical journalism in these islands? Shall we ever see a journal in which every page records good work by capable workers, and every sentiment bespeaks honest thought by vigorous minds? Or must it be our doom all our lives, seeking for the grain of wheat, to have to hunt through those heaps of journalistic chaff?

I expect no more than has been. I will speak only of my own department of practice—surgery,—and I will quote as an instance the *Transactions of the Royal Academy of Surgeons of Paris* as they appeared a hundred and more years ago. If all the societies, and all the associations, and all the colleges of all the English-speaking medical men (and women) in Europe, Asia, Africa, and America were at the end of the nineteenth century to give to the world a few such volumes as these few workers in France gave it at the end of the eighteenth century, I should be content. After a hundred years, with all our opportunities, we have seen no such work as this—so good, so abiding, so thorough. Why is this? Is it that the standard being lower the whole work suffers? The standard must be low when it is in effect the voice of the multitude, the behest of a "wide circulation." Are we, then, with our claims and our motives, to work down to this standard? or shall we not rather seek to create a higher standard—a standard with no upward limit, but with a very decided lower one? Shall we ask our brethren to raise their intellects to our literature; or shall we continue to make our literature easy for our brethren's brains?

Literature made easy is literature easily made. Journals are numerous, pages must be filled, and writing must be encouraged. On the same platform, with the same audience and for the same time, any one man may talk as long as any other. In order that he who runs may read, it would seem as if the writing were done running. All may write; all may speak; and all *must* read, and listen. Surely our medical Republic of Letters is the freest and openest of all. If Letters could keep wisdom alive, surely *we* are the people and wisdom will die with *us*.

The pursuit of the practical may sometimes be relieved by an indulgence in the pleasures of imagination. In this matter of medical journalism I have, I fear, been dreaming dreams. I have been imagining an Academy of British Medicine, whose Archives should contain all that is best of the work of the physicians and surgeons in Great and in Greater Britain; whose Transactions should go forth to the whole world stamped with the authority of our real leaders and masters. I have been thinking of a journal that despises circulation: amongst things that circulate this would be as gold to bronze, circulating less because it is worth more. Journals that deal not in advertisements we already have—all honour to them;—my imaginary journal should be one more such.

And so on our imagination leads us till we are pulled up by the suggestion of the wherewithal—cash. We must have gold. Whither may we look for it? We naturally think of our rich corporations and colleges and universities. Their coffers are not easily opened for purposes of extraction; for purposes of attraction, as we, when students, knew only too well, they open readily enough. The advancement of our sciences and arts ceases with our colleges when the student has paid his last fee; thereafter the student of science must be self-supporting. True, there are faint glimmerings of the dawn of brighter days for scientific research in medicine; not unreasonably may the handmaiden of research, Literature, look to her friends for oil to her lamps.

If our chartered friends refuse to help us, we might try to help ourselves.

The British Medical Association has some 17,000 members, who pay some £17,000 annually for its *Journal*. The *Journal* earns by its pages hired out for advertisements £15,000 a year. It is easy to infer how many pounds annually are at the disposal of the British Medical Association.

Now I am not going to suggest that the *British Medical Journal* be done away with. Far from it. I would encourage its growth on its own lines. Its leaders and leaderettes; its chronicles and cases, and lectures and news, and politics; and discussions on etiquette and rank and titles, and so forth, are all charming and indispensable. It is our very own society journal. Let it continue, and we shall all continue to take it in, and tell our stories in it, and air our grievances in it. Let it circulate, that it may get advertisements and we may get the money. It is scarcely a case of letting "the jingling of the guinea help the hurt that honour feels." It would be an example of devoting some of the money earned in wholesome and useful trading to the promotion of the highest intellectual interests of our profession.

What a subsidy would £10,000 a year, or even half of it, be to a journal of medicine! Think on the talents that might be gathered round the editor's chair with that sum: the artists, the engravers, and the printers! The *Transactions of the Royal Academy of Medicine in Great Britain* published yearly, or the *Journal of British Medicine* published monthly, would appear in garb worthy of itself. At the editor's table would sit a jury of the ablest men in our profession, whose fiat as to acceptance or rejection of papers would be received by all ungrudgingly. To have his paper accepted would be to the writer the highest honour; to have it rejected might stimulate him to try again. All the best work would be bound to go to it; none indifferent or bad would be received. It would be the accepted medium through which all that is best in our British medical work would be gathered together and given out to us and to the world. It would be the rival of no other journal; it would start at once above and outside of all other journals. To read it, and it alone, would be enough to keep us abreast of all new knowledge that is true knowledge. For this it need not be large: a thousand imperial octavo pages would well hold all that is worth preserving in any year's work in practical medicine and surgery.

Am I to be met with the habitual query of our time: "Would such a journal pay?" It would not pay: it ought not to pay; certainly it ought to make no profit. It might not even be able to recompense its mechanical workers; here our association *Journal* might step in and help. It would seek for no profit, and it would bring profit to no man save in repute, and to no project save our art of healing. As the years roll on it would become a treasure-house of knowledge, garnered ripe, and husbanded with loving care. The treasure in its coffers could not be bought, and could not be sold; the filth of minted lucre would never have touched it. By giving of our best to it, and by freely giving it away, we should be raising our art and enriching our brethren, and doing good to mankind.

Can we not erect such a treasure-house of knowledge for British Medicine? a place where our great men may pile up the rich products of their minds; a home for the noblest works of our native art; a museum whither those who follow us may turn, knowing that there is housed all the best craft of all the best craftsmen in medicine who are of our race. This would be our Æsculapian temple; built of thought, more durable than stone. Here the votive tablets on the walls would record only the victories of heroes over disease. There would be no space for the talk of the common people over patients; for we, who are above and independent of all mundane creeds and motives, know that one hero may raise us higher than ten millions of the common people. Let us give our heroes opportunity in their lives, and space in our temples. And before we place in the niches over the high altar the images of our true gods—the gods of Genius, and Labour, and Truth,—we shall have a burning in the market-place of the images of our false gods—the gods of Profit, and Chatter, and Puff.

I have said my short say as your president *ex cathedrâ*. I should like now, as your comrade and gossip, to take you by the lappets of your coats and whisper in your ears.

We, of the Bristol Medico-Chirurgical Society, have plenty of life in us: our life may almost be considered exuberant. To get the most good out of our vitality we must direct and control it, and perhaps even restrain it.

To our younger friends I should like to say these words: Give us your best and only your best. Speak only when you have something to say *of your own;* we care more for your spoken words than for the written words of any savants. Be loyal to us; fail not to tell us of any good thing that comes your way. And remember that in all art, the study from the life is the best.

To our older friends I should like to say: Come to us, listen to us, and speak to us. It stimulates and flatters youth to have age listen; it steadies enthusiasm to have experience criticise.

To all of us this further advice: Be brief. To this end you must diligently prepare your material; write it out; if you like, get it up by heart. Extempore talk, except in the rarest of cases, is liable to be at once diffuse and wanting in fulness. Fluent speech and concise truth rarely go together. Speak out your beliefs boldly and manfully. Make no effort to be learned or elaborate; but tell the truth as you feel it, simply, plainly, honestly, and with intelligence, and we, who are human as you are, will appreciate and understand it.

---

